# From perceptual organization to visual illusions and back

**DOI:** 10.3389/fnhum.2022.960542

**Published:** 2022-12-08

**Authors:** Baingio Pinna, Daniele Porcheddu, Jurgis Skilters

**Affiliations:** ^1^Department of Biomedical Sciences, University of Sassari, Sassari, Italy; ^2^Department of Economics and Business, University of Sassari, Sassari, Italy; ^3^Laboratory for Perceptual and Cognitive Systems, Faculty of Computing, University of Latvia, Riga, Latvia

**Keywords:** shape perception, perceptual organization, Gestalt principle of similarity, principle of accentuation, visual illusion

## Abstract

In modern vision science, illusions are compelling phenomena useful as tools to explore vision under limiting psychophysical conditions. Illusions manifest at least two issues that challenge scientists. The first issue is related to the definition of illusion and to the complexity of the mismatch between the geometrical/physical and the phenomenal domains. The second issue concerns two different meanings of the term “illusion,” respectively related to the demonstration of the illusion through the mismatch between domains and to the phenomenal illusoriness, i.e., the perception of something having the nature of an illusion, unreal, ambiguous, fallacious, and deceptive. In this work, we explored the notion of illusion starting from the principles of perceptual organization as described by Gestalt psychologists. On the basis of several phenomenal conditions, step by step, we suggested some new hypotheses, whose purpose was to answer the following questions: what is physical and what is phenomenal? Is there and, if any, what is the dividing line between illusions and non-illusions? Is it true that illusions are rare phenomena? Why do illusions exist? What is their perceptual and evolutionist role? These questions and the related issues were phenomenally discussed by deepening and extending the notion of perceptual organization and by exploring the biological implications of both illusions and illusoriness. On the basis of our results, the perception of illusion and illusoriness can be considered as a further challenge for vision scientists useful to shed new insights within the biological meanings of visual perception and within the no-man land between sensory and cognitive processes that elicit visual consciousness not fully explored yet.

## On Illusions

Historically, illusions are related to a “mismatch/disagreement” between two domains: geometrical/physical vs. phenomenal (Robinson, 1972; Coren and Girgus, 1978; Gregory, 2009; Vicario, 2011; Pinna and Reeves, 2017; Shapiro and Todorovic, 2017). This mismatch is considered as the necessary condition usually described when a new illusion is discovered and first brought to the attention of the scientific community.

Although this is a common and shared opinion mostly for astonishing phenomena, there are different and opposite epistemological positions and long-standing controversies on the meaning and role of illusions within vision science.

On one side, illusions are treated as “deviations” or “errors” in the perception of physical reality or as “discrepancies” between visual outcomes and the knowledge of the physical reality (Todorović, 2014; Pinna and Reeves, 2017; Shapiro and Todorovic, 2017). As such, they have been sometimes considered as marginal or border-line visual objects deserving little attention. At the same time and for the very same reason, illusions attract scientists whose interest is to understand the limits of vision and the way the perceptual system processes optical information. Following this line of thinking, illusions have been considered as the results of processing too much or too little information (Gibson, 1979) or arising at the algorithmic level with biases in the estimation of optic flow parameters (Morgan, 1996; Gregory, 2009).

Starting from these perspectives, the consequences are very reductive and mostly destructive, for example, by assuming that all perceptions, being deviations from the physical reality, are illusory, hence the conclusion is “if everything is an illusion nothing is” (cf. Noë, 2002; Shapiro and Todorovic, 2017; Rose, 2018). A further judgment, argument of a harsh controversy, developed several times in the history of vision science, suggests that illusions are scientifically unnecessary tools to understand both vision and also the illusions themselves (cf. Braddick, 1972, 2018; Shapiro and Todorovic, 2017). From these perspectives, illusions have been relegated to the status of niche phenomena, prodigies, and sometimes something to exhibit like freaks. More commonly, illusions have been considered as isolated erroneous phenomena, similarly to exceptions and, metaphorically speaking, as small islands within a very large ocean made of non erroneous real and true objects.

Despite and regardless of these controversies and alternative approaches, illusions continue to attract attention within and beyond the scientific community, given the mismatch between two domains that is the main engine that generates interest, curiosity, surprise, and amazement. Phenomenally, they are visual objects that require explanations; they immediately and spontaneously ask for explanations. As a matter of fact, they appear as the most prominent demonstrations of the inconsistency and fallacy of the visual system in picking up information from the visual world. As such, they represent a true scientific problem to be solved.

In summary, once discovered, they are phenomenally perceived as a problem. But this is an important issue since illusions might become useful tools to activate further visual investigations, new ways of seeing, and other kinds of deeper and, at the same time, more general ways to perceive and meta-perceive. Like paradoxes in mathematics, illusions could be useful tools to reflect on the consistency and completeness of vision and, consequently, to activate meta-perceptual processes (see Pinna, 2021).

The aim of this work is to explore the notion of illusion starting and based on the principles of perceptual organization as described by Gestalt psychologists and currently unchallenged milestones of vision not fully understood yet. New ideas, based on some prominent phenomena, will be introduced to answer the following questions: What is physical and what is phenomenal? Is there and, if any, what is the dividing line between illusions and non illusions? Is it indeed true that illusions are rare phenomena? Why do illusions exist? What is their perceptual and evolutionist role?

The complexity of these questions and of the resulting issues are here logically and phenomenally discussed by deepening and extending the notion of perceptual organization and by exploring the biological implications of both illusions and illusoriness (cf. Pinna, 2021).

## Method

### Subjects

Different groups of 30 undergraduate students (50% male and female) were involved in each experiment involving only one of the following figures. They had limited and mostly naive knowledge of visual illusions. They were unaware both of the stimuli presented and the purpose of the experiments. All subjects with normal or corrected-to-normal vision were recruited under previous informed consent in compliance with the Helsinki declaration.

### Stimuli

The stimuli were composed of the figures shown in the next sections. The size of the stimuli was about 10 × 8 deg of visual angle. Stimuli were displayed on a 33 cm color CRT monitor (Sony GDM-F520 1,600 × 1,200 pixels, refresh rate 100 Hz), driven by a MacBook computer, in ambient illumination provided by an Osram Daylight fluorescent light (250 lx, 5,600°K). Subjects were observed binocularly from a distance of 50 cm without any restriction.

### Procedure

Given the phenomenological nature of the starting questions, we addressed related issues by showing stimuli based on self-evident perceptions in different degrees (see also Verstegen, 2005; Pinna, 2010a, b, 2015; Albertazzi, 2015; Koenderink, 2015; Pinna and Deiana, 2015), instead of psychophysical measures.

Therefore the phenomenal results were planned to be as clear and prominent as possible, to isolate the qualitative attributes of principles under investigation and to test the effectiveness of the hypotheses (see also Duhem, 1906; Koffka, 1935; Popper, 1969, 1983).

The procedure was in line with the classical Gestalt one (see also Koffka, 1935; Köhler, 1938, 1947; Metzger, 1963; Kanizsa, 1979, 1980, 1991; Spillmann and Ehrenstein, 2004; Pinna et al., 2015). The first part of the procedure was based on a phenomenological free-report method, through which naive subjects reported anything they perceive in each stimulus. The second was a more quantitative method, according to which subjects were instructed to rate (in percent) the descriptions obtained in the phenomenological experiments.

#### Phenomenological task

The task of the subjects was to report spontaneously what they perceived by reporting, as much as possible, a full description of the visual outcomes. The descriptions were then judged by three graduate students of linguistics, naive as to the hypotheses, in order to get a fair representation of the subject reports. All descriptions were fast and spontaneous without time limits.

Subjects were free to make comparisons and comments, after thinking and viewing the stimuli in different ways and from different distances or positions. All the variations and possible comparisons occurring during the free exploration were noted down by the experimenter and reported if significant. This observation freedom is in line with experimental phenomenology and aimed to favor the emergence of clear and stable outcomes.

#### Scaling task

Subjects were asked to rate (as percentage) the main reports for each figure of the previous phenomenological task. At this stage, new groups of 30 subjects were asked to scale the salience (in percent) of each outcome. Their task was literally: “please rate whether this statement (e.g., “a rotated square” or “a diamond”) is an accurate reflection of your perception of the stimulus, on a scale from 100 (perfect agreement) to 0 (complete disagreement).” We report below descriptions whose mean ratings were greater than 85 across all experiments (about this procedure cfr. Pinna and Reeves, 2006; Pinna, 2010a, b, 2011, 2012, 2013; Pinna et al., 2015). The statements to be rated were based on the previously obtained reports, so subjects were not forced to rate outcomes that no one had reported before.

In the following sections, the results are incorporated within the text to aid the reader in the stream of discussion and argumentations.

## In The Beginning Is Perceptual Organization

Given the implicitly accepted definition of illusion (i.e., what is present in the stimulus pattern that does not match with what is perceived), we should first point out the following set of issues.

From a logical and phenomenological point of view, this definition implies some kind of comparison between two different perceptions, i.e., what is seen and measured on the stimulus pattern and what is perceived by the observer. On one side, the physical/geometrical pattern is perceived through a way of seeing based on some kinds of direct and indirect measurements or more simply through more analytic, detailed, or context-independent observations. This way of seeing is usually considered as the closest to the real world and to the truth. This is likely because the physical/geometrical domain is assumed as a prior, as the main reference system for the perceptual domain, i.e., the true and veridical reality opposed to the perceptual domain, that is, on the contrary, considered as a possible source of deceptions and illusions. This assumption implies doubts about what is perceived and doubts about the observer itself, about its phenomenal and conscious outcomes. At the same time, this assumption pushes the observer to further investigate and eventually correct the deception. This point will be discussed in more detail in the “Conclusion” Section.

On the other side, there are the immediate, spontaneous, and phenomenological outcomes of seeing, that we ordinarily trust except for some limiting, unclear, or deceiving conditions. They are other from the physical/geometrical results and, when they reveal differences or mismatches, then the visual system is assumed as the only one responsible for errors, discrepancies, and illusions. In short, the physical/geometrical domain is always assumed as true, while the perceptual domain could be sometimes false and the visual system is the only possible source of falsehood.

These opposite values of truth assigned to the two domains might be related by default to biological requirements necessary for survival in order to let a visual system to further investigate and decide about the truthfulness of the perceptual outcomes in relation to what is actually present outside in the real world. As a matter of fact, a hiss, an apparent movement in peripheral vision, or a shadow could be a predator or just a hiss, a trivial casual motion, or a shadow of the grass rippled by the breeze.

There is a third way of seeing, that we ordinarily experience both as scientists and as naive observers. This way arises when we compare the outcomes of the previous ways (geometrical/physical and phenomenal). This is necessary to detect possible mismatches between them. Actually, this is a true process of meta-perception, i.e., a further and following way of seeing looking down possible disagreements between the previous ways of seeing.

In summary, the notion of illusion might contain at least three different and complementary ways of perceiving: physical/geometrical, perceptual, and meta-perceptual. They are all phenomenologically experienced even though they are placed at different levels of reality (Metzger, 1975). These different ways of seeing will be demonstrated by describing and perceiving the stimuli illustrated in the next sections.

As a matter of fact, mismatches are almost always present in the visual world. However, not always we perceive illusions. Not all the mismatches appear illusory. The perception of an illusion implies the perception of something illusory that is more than a single non-illusory mismatch. In other words, it should contain some specific visible attribute that delivers what we call an “illusion.” To examine and debate the meaning of the notion of illusion we start first with conditions where illusions are apparently absent.

**Figure 1:**
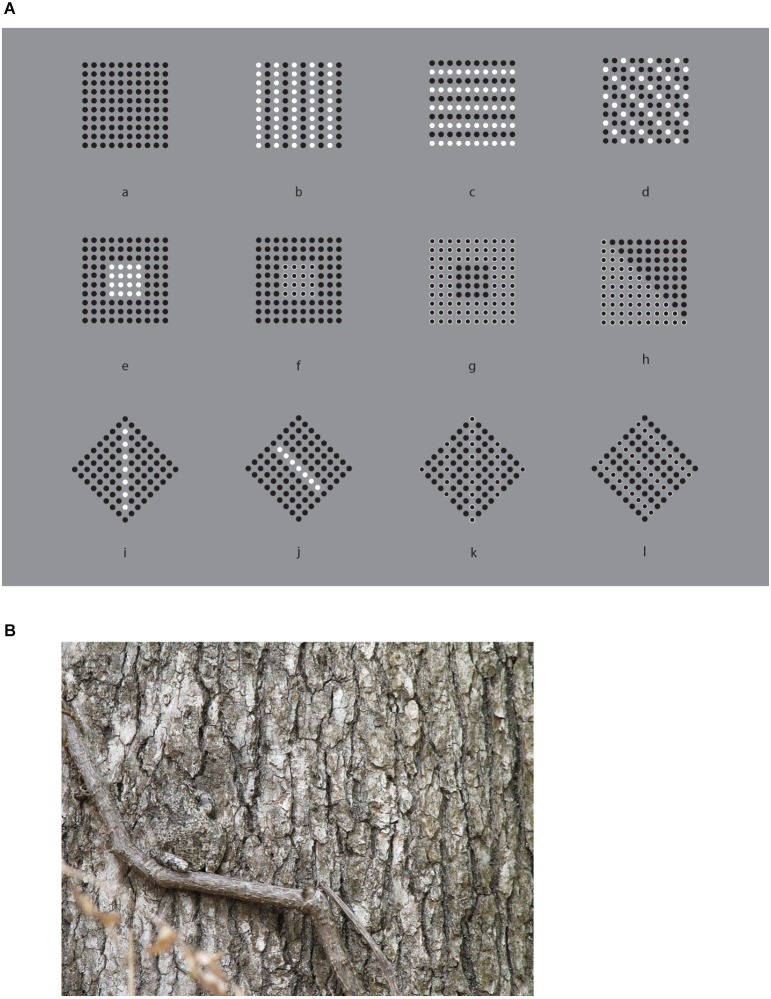
**(A)** The proximity and similarity principles: all else being equal, the closest and most similar elements are grouped together creating columns, rows, obliques, inset squares, and right triangles (a-l). **(B)** Find the frog.

In Figure 1, 12 stimuli based on the classical conditions as used by Wertheimer (1922, 1923) are illustrated. Starting from patterns like these, Wertheimer (1922, 1923) was the first to study the problem of perceptual organization in terms of grouping by discovering the following well-known grouping principles: proximity, similarity, good continuation, closure, symmetry, convexity, Prägnanz, past experience, common fate, and parallelism. Through these principles, he answered two basic questions: how do individual elements “go together” to form a holistic percept? How are wholes perceived starting from single discrete elements?

Figure 1A shows a square matrix composed of dots horizontally and vertically aligned. The dots are perceived as creating a stable square made of rows and columns. By drawing attention within the whole square, inner groupings of squared sub-matrixes are mostly perceived. Sometimes, paying attention to rows or columns, they pop up becoming alternated options mainly unstable and highly reversible. Therefore, if the rows emerge, the columns remain “invisible” and *vice versa*. Considerably more difficult or nearly impossible is to perceive oblique or diagonal groupings. Attention is for this purpose very ineffective. Other kinds of inner shapes are even more unlikely.

These perceptual results do not manifest any significant mismatch with the geometrical description of the stimulus pattern. There is no illusion. Except for the possible groupings previously described, one can say that we all perceive what is actually there. Moreover, since the vertical and the horizontal distances among the dots are closer than the oblique ones, phenomenally these results can be easily attributed to the proximity principle, as suggested by Wertheimer, stating that, all else being equal, the closest elements are grouped together.

These groupings are not perceived as illusory. As reported by the observers, they are not illusions at all. Indeed, proximity is a clear geometrical and phenomenal attribute and rows and columns are simply its expressions, a “true” consequence.

In Figure 1Aa, by reversing the polarity (from black to white) of alternated columns of dots (Figure 1Ab), the grouping by columns, previously very weak, unstable, and reversible, promptly emerge and win against rows and square sub-matrixes, now become invisible. Again, other kinds of organizations, although theoretically possible, are totally invisible or phenomenally impossible. All of them are somehow camouflaged. They might be there only if they are properly highlighted. Attention is not enough.

The claim that they are camouflaged is not totally correct and true. Perceptually they do not exist, although geometrically they are possible. Nonetheless, one can say that this camouflage is not an illusion. It is in fact very different but, at the same time, much like animal camouflage (Figure 1B) used for concealment, for example by making bodies hard to be seen, cryptic through a strong resemblance to the background, and thus totally invisible. In contrast with the camouflage of Figure 1Ab, animal camouflage is usually perceived as illusory. It is considered as deceptive, fallacious, untrue, mendacious, and misleading. In short, animal camouflage appears illusory showing illusoriness, namely a perceptual attribute not necessarily related to the presence of a mismatch, but perceived by itself as related to a sense of strangeness and oddity.

Predators “know” illusoriness and look for pray illusoriness. At the same time, prays “know” that, therefore they are continuously alerted looking for predator illusoriness. The difference between the two kinds of camouflage, respectively of Figure 1B and animal camouflage, is an intriguing issue, since they are structurally very similar, even, perfectly equivalent.

Quite simply, as in the case of proximity, in the columns of Figure 1Ab we can invoke Wertheimer’s similarity principle stating that all else being equal, the most similar elements are grouped together. The columns of Figure 1Ab are a direct consequence of their similarity. Instead, in Figure 1Aa, proximity is responsible for the same strength for both vertical and horizontal organization. This is why row and columns alternate reversibly in their emergence.

It is worth noting that, under these conditions, the similarity principle plays synergistically with the proximity operating only along the vertical grouping, thus disrupting and making invisible the rows. As such, the similarity operates like the disrupting camouflage common in animal liveries painted with contrast disorderly colorations like the frog of Figure 1B. We restate that the two kinds of camouflage do not appear illusory in the same way. Only the animal’s disruptive camouflage appears as a possible source of illusion. As a matter of fact, although the frog can be found, the rows are impossible to be seen. Therefore, the illusoriness emerges only when the trick is discovered, otherwise, the frog does not exist. This is different from saying that it is invisible.

By playing again with the similarity principle, it is now easy to switch from columns to rows, as shown in Figure 1Ac, or to obliques as in Figure 1Ad. Again, at a first glance, there is no evidence of illusion. Phenomenally these groupings do not appear as illusory but are consistent with what is actually present within the geometrical pattern.

Nevertheless, there are some well-known conditions where alike groupings are instead perceived as illusory. This is the case of the star constellations, which have been seen and coded as animal, people, or objects. The stars are grouped in constellations mostly on the base of proximity and similarity principle. The International Astronomical Union assumes 88 constellations covering the entire northern and southern sky. The names of these constellations were given mostly by ancient Greeks and Romans. Interestingly, other civilizations used to group the patterns of stars in the night sky differently. For instance, the Mesopotamians named the constellations long ago (around 3,000 BC) according to animals or human occupations. Ancient Egyptians named them after their gods and goddesses. Ancient Chinese divided the night sky into four groups, representing the Red Bird of the South, the Black Tortoise of the North, the Blue Dragon of the East, and the White Tiger of the West. In ancient India, people saw 27 divisions centered on a specific star associated with a god or goddess. It is even more interesting to highlight that aborigines of central Australia considered the spaces between the stars more important to create grouping than the stars themselves. Therefore, it is the darkness in between stars to create visual patterns. This is particularly interesting and worth to be further investigated.

Perceiving constellations is not simple and immediate. They can be easily considered as illusions with the meaning of deceptions. This is likely due to how difficult is to shape these constellations and, at the same time, to the arbitrariness and to the co-existence of a high number of possible solutions. The phenomenal attribute is not illusoriness but mostly arbitrariness. This is not the case for Figures 1Aa–Ad and of the other stimuli illustrated in Figure 1. They do not show any illusoriness since the similarity is perceived both as a geometrical and perceptual attribute.

The following stimuli are illustrated in Figures 1Ae–Al demonstrates the strength of the similarity principle in highlighting different patterns within the same square matrix. They are respectively inner squares, triangles, vertical, or oblique lines. The saliency of these emerging subsets, under these conditions, comes from the reversed contrast of the contours of the dots. The effectiveness of the contrast polarity is related to the highest luminance difference (black-white) on a gray background among different element components.

Two main phenomenal results within these stimuli are: the grouping, on one side, and the independent dots, on the other. Both outcomes can be perceived at the same time. We see that the emerging arrangements are made of unconnected and independent elements. The elements are both connected by their similarity and unconnected by their separation. Therefore, they can be perceived in both ways. Given these two opposite results, the question are: what is perceptual and what is physical/geometrical? Is grouping phenomenal or physical? What is the perceptual meaning of the term “grouping”? What are the phenomenal implications of the grouping?

Before going deeply inside these questions, what appears sure is that in these patterns it is hard to perceive illusions. The groups emerge since they are highlighted and this is both a phenomenological and geometrical-physical result. Not even in Vision Science, these kinds of results were included within any possible catalog of illusion. Rather, they are generally assumed as good examples of perceptual grouping and nothing more. In conclusion, grouping does not appear illusory; it is not an illusion. Is this true? Is this the final conclusion?

## Perceptual Organization as A Source of Illusions

Although the previous conclusions might appear self-evident and trivial, on closer inspection of Figure 1; they should be reconsidered in light of the following more intriguing results and issues. Let us try to find out whether there are hidden or under threshold illusions nonetheless induced by grouping.

### The rectangle illusion

By carefully comparing Figures 1Aa–Ac, the grouping in columns and rows of Figures 1Ab,c seems to slightly deform and reorient the whole geometrical square matrix of dots in opposite directions. More particularly, the columns induce an upwards elongation of the whole square matrix, now perceived as a vertical rectangle, if compared with the control illustrated in Figure 1Aa. On the contrary, the rows elicit the perception of a wide horizontal rectangle.

**Figure 2:**
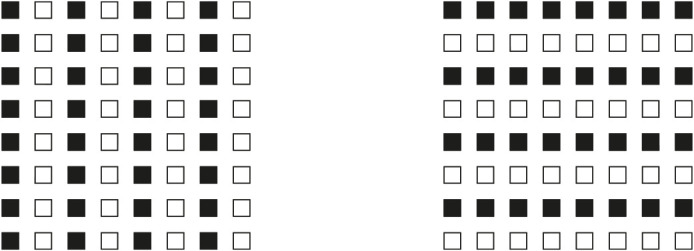
By zooming on the 3 × 3 sub-matrixes of small squares placed at the top right side of the whole groupings of squares, the columns appear to induce an upwards elongation of the sub-matrix now perceived as a vertical rectangle. The rows, on the contrary, elicit the perception of a wide horizontal rectangle.

These results are just noticeable, however, they can be more easily perceived and much stronger by zooming, drawing attention, and isolating, for example, the 3 × 3 sub-matrix of dots placed at the top right side of the whole matrix. This way of seeing can be made easier and the results more prominent in Figure 2, where the element components are now composed of small squares (cfr. Pinna, 2010a, 2011).

There is a further just noticeable phenomenon nested within these outcomes. By examining the shape of every single small square, it is not difficult to see the same kind of distortion imparted to the whole matrix. Therefore, every small square arranged in a column appears slightly extended in the same direction, similarly to a vertical rectangle. On the contrary, the square components of the horizontal grouping are perceived as enlarged as horizontal rectangles (see also Pinna, 2010a, b, 2011). This nested phenomenon will be demonstrated in greater visibility in Figure 20B.

**Figure 3:**
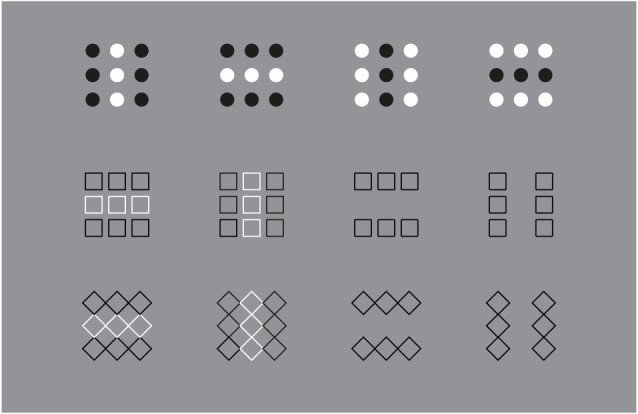
The global geometrical squares, made of dots, squares, and diamonds, appear as horizontal or vertical rectangles according to their horizontal or vertical grouping due to the similarity or proximity principles.

Similar or even stronger global deformations are perceived in Figure 3 (first row), where analogous sub-matrixes of Figures 1Ab,c, made up of 3 × 3 dots, are shown. Global elongated vertical rectangles are reported when the grouping is vertical and large horizontal rectangles when it is horizontal. Accordingly, the four global geometrical squares (second row), made up of square checks, are seen as horizontal or vertical rectangles according to their horizontal or vertical arrangement related to similarity (left) or proximity (right) principles. Likewise, whole shape distortions are seen by replacing squares with diamonds (third row). Also, the single squares of the second row can be easily perceived as illusory and distorted in the same direction of the whole grouping.

Under these conditions, the answer to the question “are these results visual illusions?” is mostly affirmative. The perceived illusions demonstrate that grouping principles affect not only the way elements in the visual field “go together” to form integrated, holistic percepts, but they also define the whole shape by imparting directions and, consequently, deformations along the emerging orientations.

The previous results highlight some basic issues that are worth mentioning. First of all, vision is demonstrated to be a sensory mechanism adapted to pick up information at different levels of details and complexity. It operates through different ways of seeing able to detect different sources of information (e.g., elements unconnected by space separation and connected by similarity) and visual shape meanings placed at different levels of perceptibility (e.g., shape deformations and other attributes to be described in the next sections). Moreover, the levels of perceptibility include not only those popping up immediately but also those just noticeable and implicit, and even those totally invisible at a glance (e.g., the rectangle deformation of the single checks).

This is especially true in predator-prey interaction, where the ability to pick up different kinds of information, to use different ways of seeing and to explore different levels of perceptibility is essential for survival. Implicit or “invisible” outcomes can be crucial, therefore a successful living being should possess the ability to detect them.

A second remark that is a corollary of the previous, is related to what we call “gradient of phenomenalness,” according to which visual information is distributed along a gradient of phenomenal evidence depicting different layers of perceptibility, whose highest position (the top) is taken up by the most prominent and immediate outcome, while the lower layers contain more and more implicit results still perceptible under specific ways of seeing. At the bottom, are expected invisible but even possible outcomes. A scientific investigation of this gradient could be useful to understand different levels of consciousness, how vision plays and moves within the layers of the gradient and how visual information mutually and spontaneously self-organize on the basis of biological and computational constraints.

It is reasonable to think that Darwinian fitness, i.e., the relative ability of an individual (or population) to survive, reproduce and propagate genes to the next generation, is related to the ability to move and explore the gradient of phenomenalness in order to pick up crucial kinds of information for survival even when they are placed at the lowest layers of the gradient. The reproductive success depends also on the ability to perceive the attribute of illusoriness and to detect by comparison different levels of realness.

On this matter, it is critical to explore the connection between illusion and reality and between illusoriness and realness. In the next sections, the connection between grouping and illusions will be demonstrated stronger and stronger by following the rationale and the following outcomes.

### From Helmholtz’s square to the illusion of element disposition

**Figure 4:**
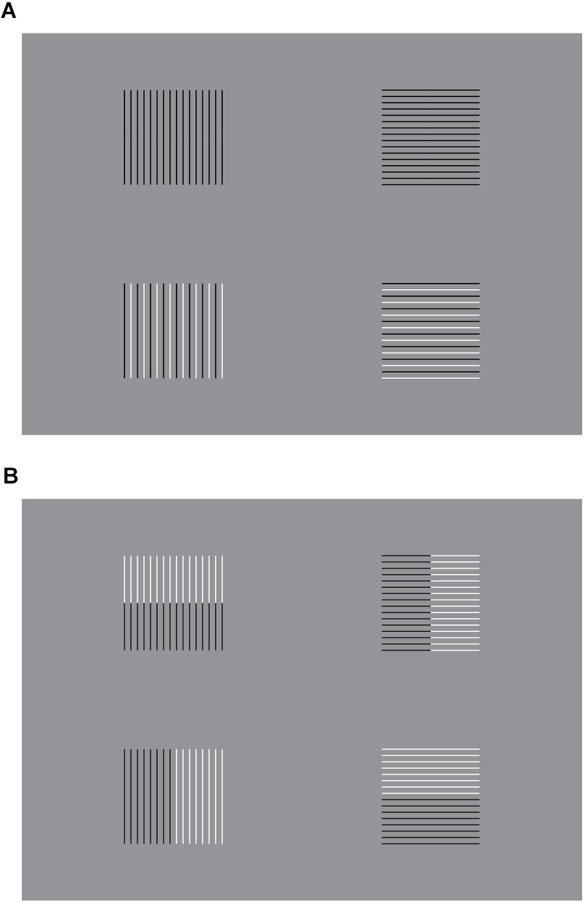
**(A)** Two examples of Helmholtz’s square illusion showing the widening of the square made up of vertical line segments and its lengthening when the lines are placed horizontally. **(B)** The size effect of Helmholtz’s squares can be strengthened (first row) or weakened (second row) by pitting the sources of directional organization, imparted by the similarity, in favor or against the disposition of elements.

The rectangle illusion described in the previous figures can be related to the notorious Helmholtz’s square (Figure 4A), according to which a whole square appears wider similarly to a horizontal rectangle when it is filled with a stack of vertical lines and higher, like a vertical rectangle, when filled with horizontal lines (Helmholtz von, 1866; see also Da Pos and Zambianchi, 1996; Pinna, 2011).

By comparing Helmholtz’s square and the rectangle illusion, it does not go unnoticed the apparent opposite direction of the two phenomena. This appears as a contradiction since the same results are expected on the basis of a similar structure between the two phenomena. In fact, vertical groupings of elements in Figure 3 seem equivalent to the vertical lines of Figure 4A, conversely horizontal groups are the same as the horizontal lines. Nevertheless, the vertical groupings of Figure 3 elicit a vertical rectangle, while the vertical lines of Figure 4A create a horizontal rectangle. The same unexpected contradiction is reported for the horizontal groupings and lines. Hence, somewhere there must be a mistake. By realizing this mistake, human vision assumes something similar to the laws of non-contradiction and excluded the middle.

Apparently, an unsolvable antinomy seems to emerge from the two phenomena. More particularly, since a square filled with vertical lines appears wider than an identical square made up of horizontal lines, why does a square composed of dots vertically grouped in columns is perceived, on the contrary, taller and narrower than the same square with elements horizontally grouped? At the first look, the explanation of Helmholtz’s square leaves the rectangle illusion unexplained and* vice versa*. Consequently, one phenomenon can be considered as a counter example of the other from a logical viewpoint. The choice is twofold: consider the two effects as different, therefore requiring distinct explanations, or as part of the same kind of phenomena, consequently, asking for the same explanation in terms of perceptual organization. The latter choice could be useful to review and extend the notion of perceptual organization, as will be seen.

Before getting inside the solution, further reflection is needed. Phenomenally, both results appear, even independently, as illusions and even more so through the comparison of the two outcomes eliciting the detection of the apparent contradiction. Within these figures appear more clear the meaning of the visual property of “illusoriness,” totally invisible in Figure 1, and defined as the phenomenal attribute of something having the nature of an illusion, something unreal, ambiguous, fallacious, and deceptive. This general and apparently tautological definition will be specified and declined through the following stimuli.

Under the previous conditions, the illusoriness mostly emerges after the perception of two kinds of mismatches: one related to the geometry of each whole shape compared with its perceptual result, the other due to the apparent antinomy between Helmholtz’s square and the rectangle illusion (The illusoriness will be shown much more prominent in the next figures.).

The question is now: can the two apparent opposite phenomena be predicted by the same explanation? Superficially, the answer is negative. To become positive the contradiction should be solved (cfr. Pinna, 2021).

A deeper and more analytical inspection of the kinds of arrangement of elements in both illusions reveals that there are at least two sources of dispositions. One is local and belongs to each element component: dots in Figure 3 without any main direction and line segments in Figure 4A with clear specific directions. The other source is global and related to the direction of the disposition/juxtaposition of elements of the first source.

Therefore, the local directional source within Helmholtz’s squares belongs to the lines, vertical or horizontal. However, the global directional source, imparted by the disposition of the elements in stacks is the opposite, horizontal and vertical. In short, vertical lines are distributed horizontally, while the horizontal lines, vertically. But the outcome of the second source is exactly what is perceived in Figure 3, where the grouping of the dots of the second source is the same as the lines in Helmholtz’s squares, namely, the dots are arranged horizontally or vertically analogously to the lines of Helmholtz’s squares.

Now the conclusion is simple: since the illusions are based on the whole shape made up of different elements, then the solution to the apparent antinomy depends on the equivalent disposition of elements that create the whole shapes. The global grouping is responsible for the whole perceived shape either when the element components are lines or dots. Therefore, the Helmholtz’s square filled with vertical lines is perceived as wider because of the horizontal disposition of lines, that is equivalent to the horizontal arrangement of dots of the rectangle illusion. Conversely, the square made up of horizontal lines appears slimmer because of their vertical disposition in a stack analogous to the vertical grouping of dots. This solution satisfies the laws of non-contradiction and excludes the middle and makes possible further extension of what we can call “illusion of element disposition” that imparts a significant deformation of the whole geometrical square in the same direction of the disposition of its elements.

It is worthwhile pointing out that likewise in magic tricks, the unveiling of the tricks of a magician brings to the destruction of the mystery. When truth irrupts the astonishment of the magic, the mystery is killed and what remains is just a simple real fact, the trick. A similar attitude is experienced when the frog of Figure 1B is discovered and in all circumstances where a prey discovers a predator and* vice versa*. In our conditions, the solution of the contradiction weakens the astonishment of the illusoriness and the illusoriness itself. However, it can be restored through the following new variations and “tricks.”

A first corollary deduced by the solution hypothesis of the contradiction is a simple way to significantly enhance or reduce the strength of the rectangle illusion and Helmholtz’s square by playing with the grouping principle of similarity, as illustrated in Figure 4B.

In the first row of Figure 4B, the outcomes of Helmholtz’s square illusion are clearly enhanced, since two horizontal (left) and vertical (right) groupings/dispositions operate synergistically. More particularly, the two groupings are related to the juxtaposition or stack disposition of elements and to the whole configural organization created by the similarity. They are both horizontally (Figure 4B-left) or vertically (Figure 4B-right) oriented. By comparing the two geometrically equivalent geometrical whole shapes, made of line segments, they appear dissimilar in size, much more than the two Helmholtz’s squares of Figure 4A, where, even if a difference is perceived, it is much weaker than the one of the variations within Figure 4B-first row.

On the contrary, in Figure 4B-second row, the effects are reduced or totally annulled, since the two groupings are now pitted one against the other. The resulting effect is even weaker than the one perceived in Figure 4A.

The previous figures demonstrate that similarity and juxtaposition impart a direction to the element components of a grouping and as a consequence a shape deformation along the same direction. We can extend this statement through a second corollary stating that the addition of any kind of inner directions in favor or against the two previous groupings can respectively increase or reduce the whole shape deformation along that specific direction. The corollary can be easily demonstrated throughout the following three figures.

**Figure 5:**
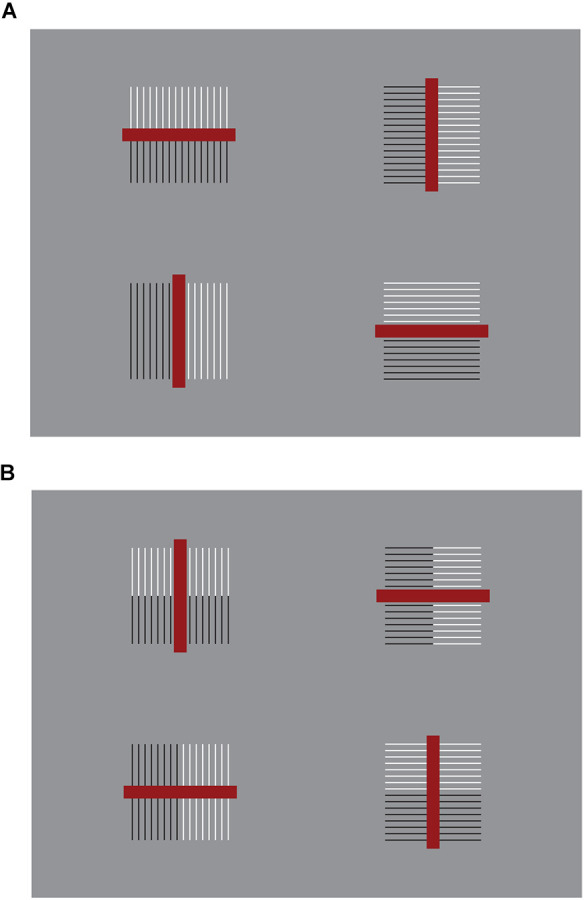
**(A)** A rectangle, horizontal or vertical, superimposed upon Helmholtz’s squares, enhances (first row) or reduces the resulting effects. **(B)** A rectangle orthogonal to those of **(A)** reverses the results.

In Figure 5A, a rectangle, horizontal or vertical, has been superimposed upon the stimuli illustrated in Figure 4B in order to enhance their effects. On the contrary, in Figure 5B, the rectangle is placed orthogonally to reduce the effects.

The results clearly support the second corollary. They are more effective and prominent by comparing the outcomes of Figures 5A,B. Moreover, Figure 5B shows noticeable effects by comparing the differences between the stimuli placed on the left and right or on the top and bottom.

It is important to point out that the directional effect of the rectangle increases by increasing its dissimilarity with the surrounding elements, e.g., by changing the color or even other attributes (not illustrated).

**Figure 6:**
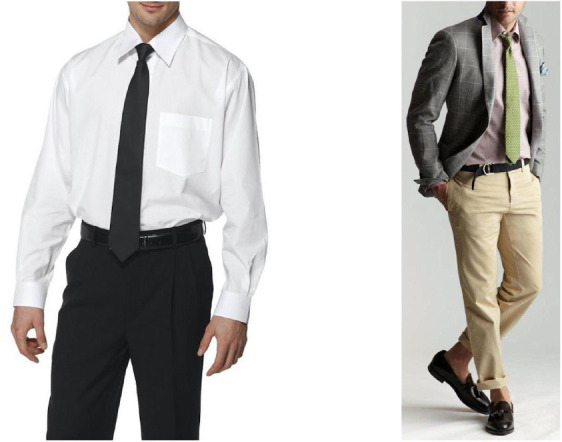
The role of a necktie could be related to the elongation effect imparted by the red rectangle of the previous figures.

It is not unreasonable to hypothesize that the common use and the aesthetic role of the necktie could be related to the elongation effect imparted by the red rectangle of the previous stimuli (Figure 6). A significant effect was demonstrated in alive men with and without a tie. It is notable that usually, the color of the ties is very different from the one of jacket, blouse, and trouser. This is consistent with the dissimilarity of the rectangle as inducer of directionality as shown in Figures 5A,B.

These notes suggest that there are aesthetic or social traditions, trends, and fashions that could be related to visual phenomena not actually conscious but visually successful like the tie or other cloth accessories. Aesthetic appreciation and empirical aesthetics dealing with the nature of beauty and taste could be basically reduced to more simple visual attributes like the “tie effect” belonging to the red rectangle.

It is reasonable to hypothesize some kind of natural selection in the evolution of fashion based on their visual and aesthetic effectiveness. A sort of Darwinian fitness in fashion might denote a quantitative representation of the individual reproductive success of a cloth. This reproductive success can be measured in terms of the amount of spreading within the human population, duration of survival, and number and quality of variations and further evolutions. It can be likely possible to talk about survival of the fittest interpreted as the survival of the form that will leave the most copies of itself in successive generations. We will be continuing this discussion in the “Conclusion” Section.

The previous stimuli demonstrated a deep connection between perceptual grouping and visual illusions. Under these conditions, there is no break of continuity between the two. Visual illusions seem to depend on visual grouping. In other terms, illusory phenomena can be reduced and explained in terms of interactions between simple principles of perceptual organization.

This hypothesis also works the other way around. Illusions are useful to better understand the perceptual organization, as shown in the case of the illusions of element disposition, that is something new within the domain of the known Gestalt Principles.

Moreover, the so-called principles of grouping not only put together elements creating wholes separated from other wholes but elicit different directions, segregations, orientations, deformations, instabilities, locations, and also polarizations that, as we will demonstrate step by step, can give rise to apparent motion. Therefore, grouping is not just grouping but much more.

The previous outcomes imply that perceptual grouping, even in the most simple conditions, is always accompanied by satellite effects that can power up illusions and illusoriness in both single element components and wholes.

These further derived attributes are triggered by the principles not restricted to the Gestalt ones as shown in Figures 5A,B. In addition, none of the known principles can describe the role of the red rectangle that operates like an accent that emphasizes and highlight a specific direction that, in its turn, induces a shape deformation. As previously suggested the red rectangle is something totally dissimilar from the other elements, therefore not attributable to the similarity principle, and for this very reason it influences the other components of the stimulus. The main role of the accentuation is based on the dissimilarity and on the location within or nearby a shape.

To get a better understanding of the satellite phenomena of perceptual grouping, of visual illusoriness, and of the role of the accentuation principle the next stimuli might be useful.

### Shapes from accentuation: the diamond-square illusion

The tie effect of the rectangle, not only elongates a whole pattern of elements but it can also accentuate one or another phenomenal shape within the same geometric shape. In other words, it can make visible highlighting or switching one or another shape within a set of possible shapes included in the geometrical shape. The geometrical shape is only one, but the phenomenal shapes are more than one, as we are going to prove.

To clear up this point, let us think of a square as a regular quadrilateral made of four equal sides and four equal angles. Phenomenally, these simple components define and contain the complexity of what we call “square.” As a matter of fact, these elements manifest different or even opposite properties. The sides show flatness, while the angles appear pointed. Now, let us think first of a square as it is usually represented and imagined (see Pinna, 2021), namely with the sides oriented along the main direction of space, i.e., vertically and horizontally. Afterwards, let us think of the same geometrical shape rotated at 45°. At 0° and 45°, indeed, they are perceived as two different “shapes.” The first is a “square,” and the second, a “diamond.” The square appears flat placed on one of its sides, the diamond pointed and seated on one of its angles.

The use of two terms to name the same geometrical shape, simply rotated, represents significant evidence of the presence of two different shapes within the same geometrical object. This implies that the geometrical square holds at least two different and possibly opposite phenomenal shapes: a square and a diamond.

This thought experiment is necessary but not sufficient for a full set of demonstrations. The demonstrations we are looking for should be related only to the accentuation due to an induced direction or, more generally, to the principle of similarity that is our main target. Finally, our demonstrations should appear immediate and quite phenomenally evident.

**Figure 7:**
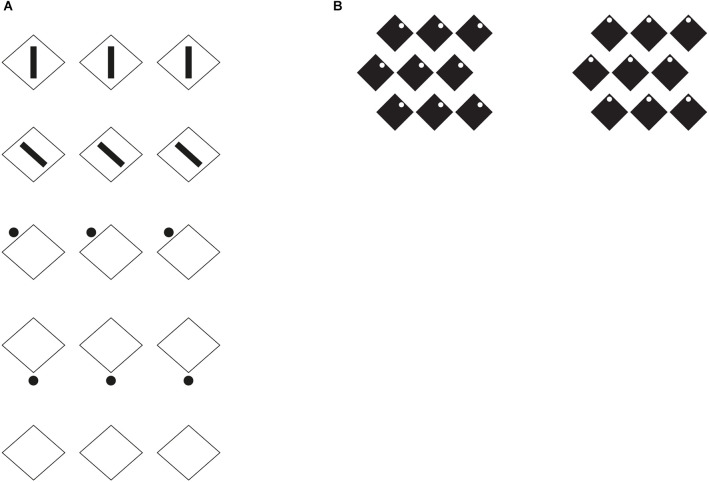
**(A)** Diamonds (first and fourth rows), rotated squares (second and third rows), control (fifth row). **(B)** Rotated squares (left), diamonds (right).

In Figure 7A, the first row shows three geometrical squares oriented like diamonds, whose inner rectangles accentuate their appearance as diamonds. Compare this result with the control of the last row.

Here, the presence of three aligned diamonds induce the configural orientation effect, i.e., the perception of local spatial orientation determined by the global spatial orientational structure studied by Attneave (1968) and Palmer (1980). This effect is expected to highlight only the pointedness along the three diamonds. The pointedness is further accentuated by the inner rectangles of the first row oriented along the two poles. In the second row, the inner rectangle is rotated 45° anticlockwise. As a consequence, the same checks of the first row are now perceived more like rotated squares. This outcome wins against the configural orientation effect. The rotation of the rectangles along a direction orthogonal to the sides accentuates the sidedness of the shapes instead of the pointedness, hence, eliciting the perception of squares.

The third and fourth rows of checks demonstrate similar accentuation effects imparted by single dots placed nearby the two components of the checks. More particularly, in the third row rotated squares are perceived, while diamonds emerge in the fourth. It is worth mentioning that when perceiving the rotated squares a significant number of naive subjects have turned their head to better perceive the square. The head rotation is like an adjustment or an adaptation to the perceived result, thus bringing back the rotated square to the iconic square without rotation (cfr. Pinna, 2021), i.e., with the sides vertically and horizontally oriented. This effect on the observer can be more easily produced by comparing the two conditions of Figure 7B.

There are at least two main ways of seeing the stimuli of Figure 7A. In one way, we perceive that the checks of the first and of the second row are geometrically equal, nevertheless, in another way we see the difference between the two. The terms “pointedness” and “sidedness” are useful to describe the specific differences. However, the names of the two shapes, “diamonds” and “rotated squares” is more iconic and it is phenomenally what was to be demonstrated (QED). Obviously, we perceive that diamonds and rotated squares are the same shapes, nonetheless, they appear different.

A third way of seeing compares the results of the local and analytical view with the holistic one. In other words, we perceive that physically they are the same checks but we also at the same time through the comparison, see that they are different shapes. It is this contradiction that triggers the illusoriness of the stimuli, and the illusoriness brings perception to a higher level of consciousness through the meta-perception of the outcomes of the two different ways of seeing. In other words, these results could suggest the emergence of consciousness as a result of the perception of illusoriness.

This is a heavy statement that requires a much stronger demonstration that we will acquire step by step. For the moment it might be sufficient to think of a prey, for instance a gazelle, grazing in pastures of savanna, continuously alerted by sound, movements of frasche, shadows, and smells. These surrounding stimuli could be perceived as background safe noise or as dangerous and life-threatening clues of an ambush or of the imminent attack of a leopard, hyena or cheetah. These possible outcomes are the results of at least two different ways of perceiving.

First of all, a basic assumption suggests that the perceptual system of the gazelle should be set up so as to prevent being always alerted and endlessly running away but, on the contrary, and at the same time, to prevent and avoid true signs of predator behaviors. Therefore, the dilemma is to avoid both false alarms with never-ending escape and inaction before a true danger is predated. The solution to the dilemma is related to the perception of the degree of realness and illusoriness perceived within the incoming stimuli. Illusoriness and realness are precisely the outcomes emerging from a comparison of different ways of perceiving and the main source of consciousness of the gazelle. Consciousness is something theoretically required. It is the necessary and maybe sufficient outcome of different ways of seeing producing different or even antinomic outcomes. Under these conditions, consciousness can be considered as a way of seeing, that compares different results within the same stimulus in order to assign a true-false value necessary to decide to escape or to remain grazing the grass.

These reflections are even more valuable if we rethink the same dilemma from the point of view of the predator, e.g., the leopard. Its behavior should be illusory and, at the same time, sufficiently true. The leopard should be conscious that its ambush must be camouflaged to be effective. Therefore, the ambush should be a true illusion, with a high degree of realness, showing or appearing as something real, for example, similar to background safe noise like branches blowing in the wind. The illusion generated by the predator should manifest a strong attribute of realness. If it appeared illusory, i.e., with the attribute of illusoriness, then it would alert the gazelle. Again, the illusoriness and the true-false antinomy are the basic perceptual components related to the consciousness of different ways of seeing.

Within the previous examples, perceptual consciousness is some kind of meta-perceptual way of seeing, to be learned, refined, and improved to ensure survival. Gazelles and leopards learn during their life, their successes and failures to improve perceptual consciousness and as a consequence actions, reactions and decisions to external stimuli.

Going back to Figure 7A, our demonstration is not complete yet. With these stimuli, we are missing the logical and phenomenological link between the classical grouping principles and the notion of illusion, which is the main purpose of this work. Through the stimuli of Figure 7A we cannot deduce that the interaction between rectangles and checks represents a case of grouping. Phenomenally the elements group together and clearly influence each other, however, they cannot be reduced to the classical Gestalt principles but to a further principle of grouping based on the accentuation of inner shape attributes and directions (see also Pinna and Sirigu, 2011; Pinna et al., 2018).

Through the next stimuli, we demonstrate the close connection between this illusion and the grouping principles. More generally, we assume that grouping principles can also be principles of accentuation according to which by highlighting specific attributes of a shape and creating directions within groups, they deform whole shapes and accentuate inner shapes within a shape.

A preliminary demonstration of the role of the similarity principle is already included in the third row of Figure 1, where the matrixes of dots, including lines of dots with reversed contrast, are perceived as diamonds when the white dots run from an angle to the other placed at the antipodes or as rotated squares when they run from one side to another.

These results were at first invisible, unnoticed, and under threshold when Figure 1 was described by the observers. They belonged to the lowest levels of the gradient of phenomenalness. Now they are perceived near the top of the gradient taking consciousness on the basis of the illusoriness highlighted in the previous and even more in the next figures. This suggests that the gradient of phenomenalness is not fixed and static but very dynamic and ready to change rapidly in relation to new ways of seeing, comparing, and new outcomes. The same thing happens for the related perceptual consciousness.

**Figure 8:**
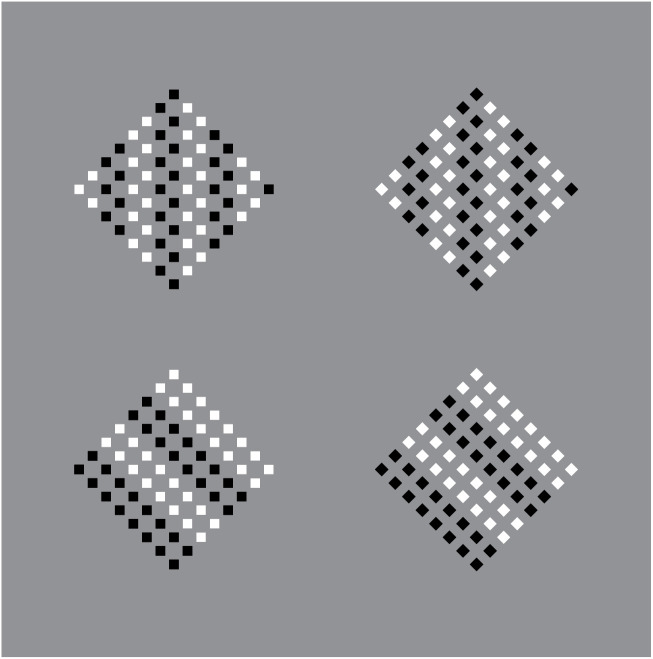
Squares and diamonds perceived as squares and diamonds (first row); squares perceived as diamonds and diamonds perceived as rotated squares (second row).

In Figure 8, we played again with the similarity among the checks inducing organizations along vertical (first row) or oblique (second row) directions of the whole pattern (cfr. Pinna, 2021). The direction of the grouping highlights, respectively, sides and angles (first row), and angles and sides (second row) of each check. As a consequence, in the first row, squares appear like a square (left) and diamonds like diamonds (right), while in the second row, squares appear like diamonds (left) and diamonds as rotated squares (right).

It is worth being aware that, although the illusion is present in the elements of both rows, the illusoriness emerges only in perceiving the shapes of the checks of the second row, i.e., where there is a clear contrast between what is seen to be there and what is perceived, i.e., squares vs. diamonds and diamonds vs. rotated squares. Contradictions and conflicts between outcomes of different ways of seeing are responsible for the emergence of the phenomenal attribute of illusoriness.

The direction imparted by the grouping influences not only the single elements but also the whole group of checks, as in the third row of Figure 1, thus, highlighting angles (first row) and sides (second row) of the whole patterns. Therefore, the whole patterns of the first row are perceived as large diamonds, while those of the second row are like rotated squares.

**Figure 9:**
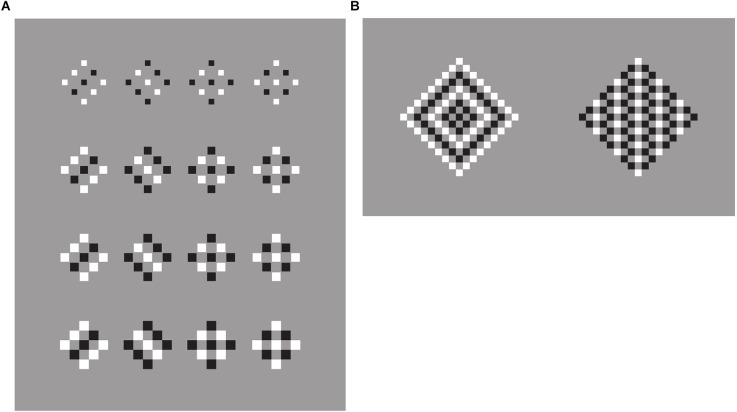
**(A)** Diamonds on the first two columns and squares on the other two. The patterns of stimuli are all the same made of squares. **(B)** Diamonds (left), and squares (right) within the same patterns of stimuli made of squares.

These results are further corroborated and emphasized by comparing the equally oriented patterns of Figure 9A, whose elements and wholes appear very different due to the similarity principle. Along the two columns on the left, the squares are mostly perceived as diamonds. Moreover, by increasing the size of the components from the top to the bottom, the checks group incorporates portions of the gray background, thus, inducing some kind of transparency effect.

**Figure 10:**
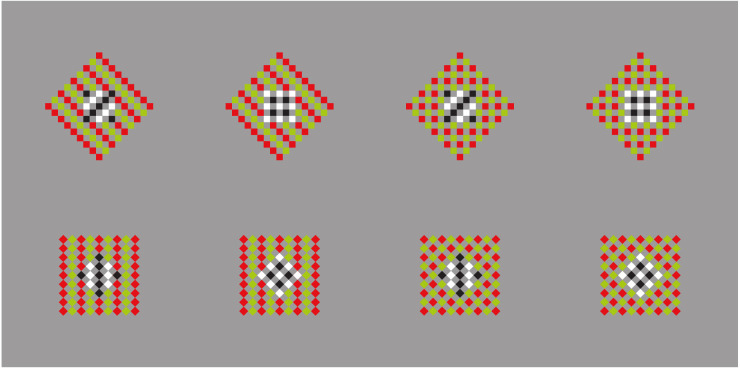
Illusory squares and diamonds within surrounding diamonds (first two columns) and squares (the other two columns) and included black and white elements made of squares (first row) and diamonds (second row).

More clearly, analogous outcomes are perceived in Figures 9B and 10. In Figure 10-second row, the global shape distortion should be noted by comparing the first two arrays on the left with the other two on the right. More particularly, the first two appear like vertical rectangles, slimmer than the other two due to the vertical organization of the diamonds vs. the oblique grouping of rotated squares.

**Figure 11:**
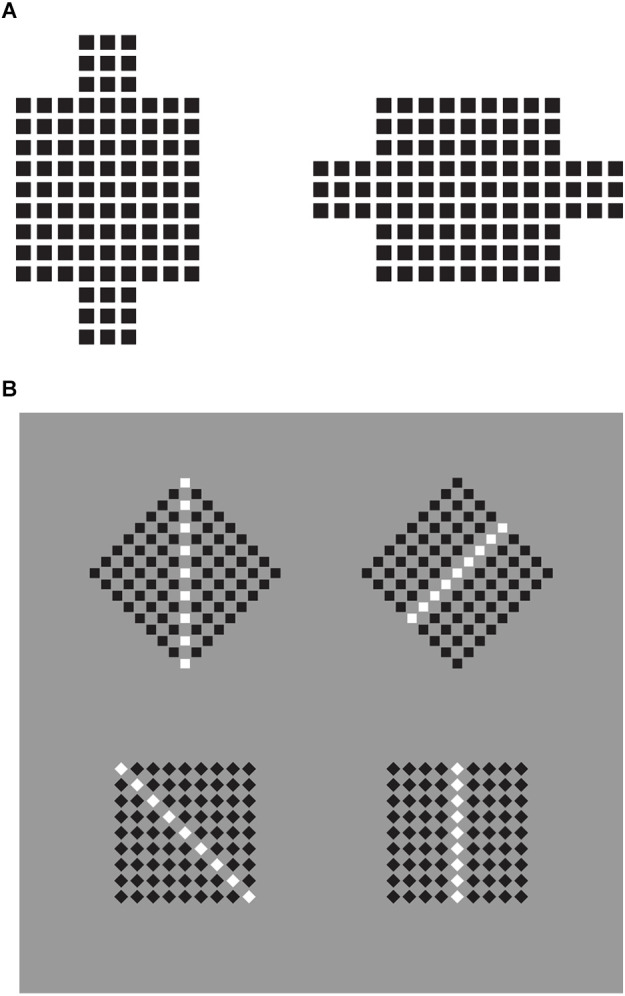
**(A)** Slimming (left) and widening (right) effects of the inner whole square made of small squares. **(B)** Squares perceived as squares and squares perceived as diamonds (first row); diamonds perceived as rotated squares and diamonds perceived as diamonds (second row).

A similar effect can be noted in Figure 11A under different conditions consistent with both the perceptual organization and the directional effect caused by the horizontal and vertical extensions. Here, the vertical organization induces an elongation and slimming effect of the whole square made of small squares. The opposite occurs in the horizontal organization. The small squares appear distorted likewise.

Together with these effects, there is a further worthwhile phenomenon to be brought out. It can be seen in Figure 11B, where the white “ties,” are perceived, in short, respectively as squares on squares (first condition), diamonds on squares (second), squares on diamonds (third), and diamonds on diamonds (fourth), induce the same effects on the surrounding checks. In other terms, the way the white elements are perceived spreads on the nearby black elements of the arrays. Therefore, if the white components are perceived as diamonds also the surrounding ones are seen as diamonds. Conversely, where they are seen as squares or rotated squares then the same effects fill in the entire array of checks.

This is in some way unexpected if we assume an explanation in terms of reference frame, according to which the larger and surrounding array is expected to impart the same kind of shape on the inner elements, on its part. Under our conditions, the causal relationship is reversed. It is the part that mostly influences the whole, not the other way around. This is supported and in some way anticipated in the light of the previous conditions illustrated for example in Figures 7A,B, where inner or outer elements define the shape of larger wholes.

Paraphrasing Gestalt psychologists, we can summarize the filling in outcomes of Figure 11B as follows: the whole is not greater than the sum of its parts. It is intriguing that the same effect can be accomplished, although less prominent, by drastically reducing the number of white elements to only one (not illustrated).

The previous results might support more and more the hypothesis of a continuum between the perceptual organization and visual illusions. However, the term “continuum” might be not totally correct since grouping principles behave like illusion generators. In fact, we suggest that these principles do not create only grouping but also a set of shape distortions and shape accentuation.

### The diagonal illusion

**Figure 12:**
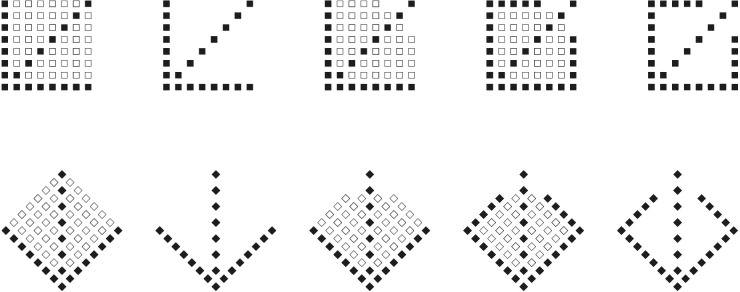
Squares and diamonds perceived as squares and diamonds (first row); squares perceived as diamonds and diamonds perceived as rotated squares (second row).

There is a further related phenomenon that can be brought to the fore. It can be observed in Figure 12. The first pattern of Figure 12 demonstrates once more the diamond-square effect already proved. In addition, there is a further phenomenon related to the lengthening or elongation of the square diagonal made of diamonds. More specifically, the diagonal appears to continue and extend beyond its geometrical end suggested by the array of squares.

This diagonal effect can be better appreciated more and more in the other patterns of the first row up to the last stimulus. This phenomenon is enhanced in the second row, where the same patterns of the first row have been 45° rotated. Now the diagonals seem to escape more saliently the geometrical convergent point of the square array of small squares. This is another source of illusoriness related to the accentuation phenomenon.

### Expressiveness from accentuation: quadrilateral and organic illusions

Previously, we showed the switch, induced by a single dot, between diamonds and rotated squares. By replacing the square with a geometrical quadrilateral, i.e., an irregular four-sided polygon, having four edges (sides) and four corners (vertices), new phenomenal shapes within the shape are expected. If a square contains at least two shapes (diamond and square), a quadrilateral with four different sides and angles should contain at least eight different shapes, one for each dissimilar attribute.

**Figure 13:**
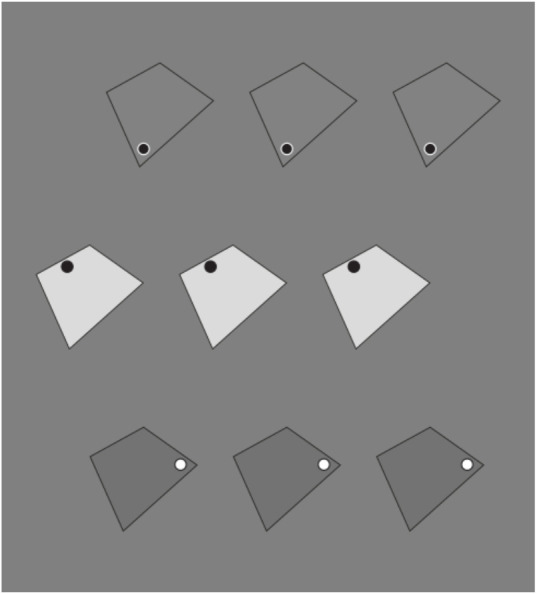
Rhomboids (first row), trapezoids (second row), a further kind of quadrilateral (third row).

In Figure 13, three of these shapes are shown in three rows where the same geometrical quadrilaterals are perceived as three different shapes: rhomboids in the first row, trapezoids in the second and a further kind of quadrilateral in the third (cfr. Pinna, 2021).

**Figure 14:**
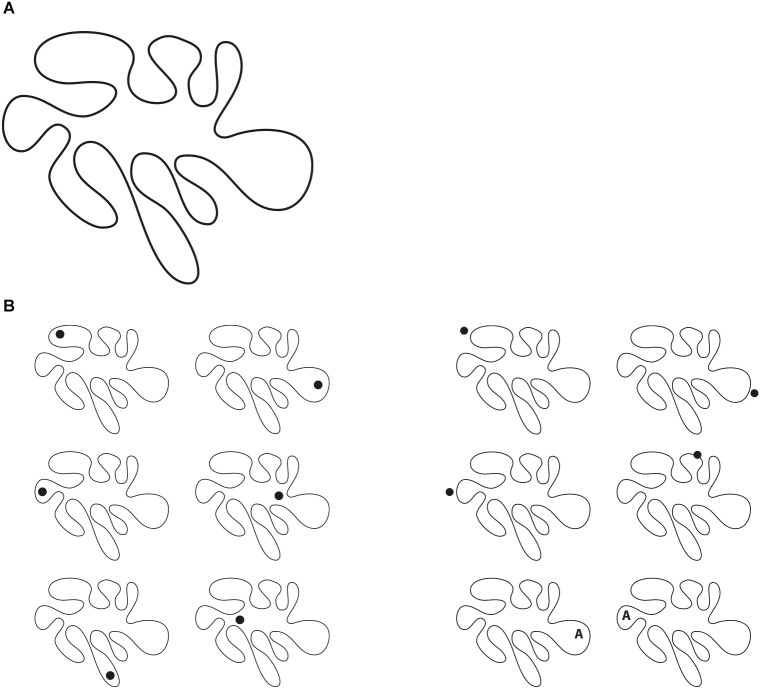
**(A)** How many shapes are included within an irregularly undulated shape like this? **(B)** Different amoeboid organisms with unique expressive attributes, related, for instance, to specific and different species with the precise position of the head, with kinds of legs peculiar to that species that appear as moving along a defined direction, just to mention a few (left). Similar results without the eye effect induced by the dot (right).

If the number of inner shapes depends on the number of dissimilar attributes of the geometrical shape, then what are the expected outcomes in an irregularly undulated shape as the one illustrated in Figure 14A? Theoretically, the number of possible shapes increases infinitely. Phenomenally most of them are very similar, however, a high number of shapes can be popped up just by adding a dot. Figure 14B-left demonstrates some of the hidden shapes accentuated by a dot placed in different locations inside the geometrical shape. The emerging shapes look like different amoeboid organisms with expressive attributes, related, for instance, to specific and different species with the precise position of the head, with kinds of legs peculiar to that species that appear as moving along a defined direction, just to mention a few (see Pinna, 2015, 2021).

These possible outcomes apparently depend on the fact that the dot can be perceived like the eye of the creature. Therefore, the eye is considered the cause of the emerging animal. This hypothesis is reasonable and phenomenally appropriate. It is reminiscent of the pareidolia, which is, in short, the tendency to perceive a familiar image in a random or ambiguous visual pattern.

However, this hypothesis cannot explain the stimuli of Figure 14B-right, showing similar kinds of creatures without the eye effect of the previous group. The main role seems to be played by the accentuation principle, which can highlight expressive qualities of hidden shapes within the shape (see also Pinna, 2011, 2021).

The question is here the same as the one asked in previous figures: are these outcomes illusions? Do they show any illusoriness phenomenal attribute? Some degree of illusoriness can be perceived. The previous discussion on the accentuation role of the dots can trigger some illusoriness, being this latter mostly a meta-perceptual property coming from a way of seeing, that compares the outcomes of other ways of seeing. In other words, we perceive that in Figure 14A there is only one shape, a single object, however, after the results of Figure 14B, even in Figure 14A, we can perceive, at the same time, many shapes with different expressive meanings. This is the way of seeing useful to instill illusoriness.

**Figure 15:**
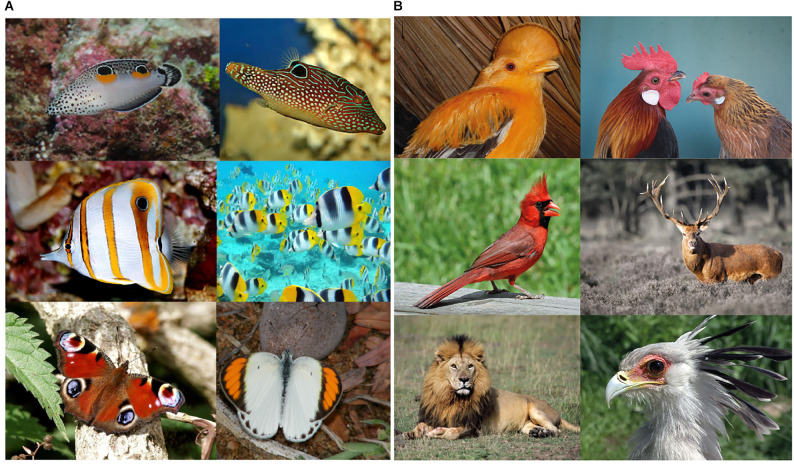
**(A)** Dots and spots painted on the livery of animal bodies in order to accentuate a multiplicity of adaptive purposes. **(B)** Crests, horns, and manes produce illusions for different biological purposes like defense against competitors or predators, courtship and mating, and social status ranking.

In nature, accents, like those of Figure 14B, have evolved in a high number of species as dots or spots (ocelli) painted on the livery of their bodies for a multiplicity of adaptive purposes (Figure 15A).

They induce illusions useful to improve success, adaptive fitness, and survival in predation, defense, camouflage, deception, mimicry, courtship, etc. Their Darwinian fitness value is significantly high by effectively playing in between illusoriness and realness.

More generally, illusions are the basic foundation of natural selection and, indeed, the true and most effective tools for survival. These illusions should be perceived and shown as real, without illusoriness, to be deceptive and, thus, effective, for instance, in the perspective of a prey. In the shoes of a predator, the ability to perceive illusoriness within illusions like these, i.e., the ability to perceive in different ways in order to discover the hidden trick and the deception, is necessary for survival.

The push-pull inter-relation between preys and predators is self-evident. In fact, if the illusions developed by preys are real, with the strongest attribute of realness, thus, fully deceiving, then predators would become extinct. At the same time, if predators were able to discover all the illusions, i.e., to perceive the illusoriness and, hence, to always reveal the trick, then preys would become extinct. It is a kind of game between predators and preys that improves abilities to perceive and develop new kinds of illusions. The push-pull game, related to Lotka-Volterra equations, also known as the predator-prey equations, is so much more complicated if we think that many preys for some species are at the same time predators for others. In addition, many of these illusions evolved at once also for courtship, sexual ritual, copulation, aggressiveness, defense, and social status. The complexity of these illusions in biology comes from the complexity of the perceptual organization, that is the true background on which grows illusions.

The complexity of the last figures extends far beyond deformations, change in size, in shape, in orientation. It involves expressive or tertiary qualities essential in real life, in biology, in human interactions within a very large range of domains, attributes, and possibilities. This is a further issue related to the previous illusions that deserves to be deepened. At this stage, we would like to show a plausible connection between the illusions previously demonstrated and the way animals and humans play with similar effects for many different purposes, not strictly associated with prey-predator interactions.

In Figure 15B, crests, horns, and manes produce effects not dissimilar from the dots and rectangles previously described. For instance, due to these add-ons and extensions, the males of these species appear taller as in our figures, but also stronger, powerful, intimidating, vigorous, healthy, and attractive. Implicit in these terms there are different biological purposes like: defense against competitors or predators, courtship and mating, and social status ranking.

All these attributes are true illusions not always real, more often used to deceive, to cheat, to bluff. They are accents used to highlight and demonstrate something more, possible shapes within a shape, to misquote previous expressions.

**Figure 16:**
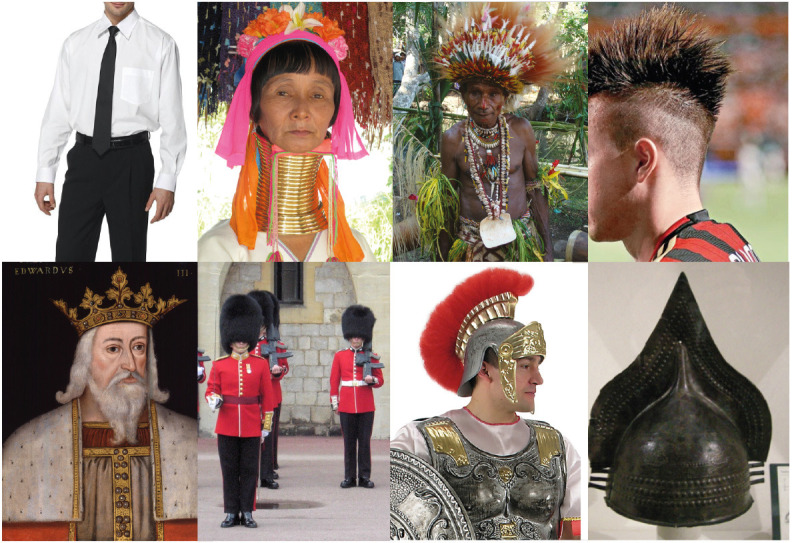
Crests and even more sophisticated illusory accessories are used by the human species for similar but even for more complex purposes.

Crests and even more sophisticated accessories are used by the human species for analogous basic purposes, as shown in Figure 16. Fashion, as already suggested, is an important player of the factory of illusions based on perceptual organization. Within the human domain, additional expressive and more complex phenomena are elicited, like all those included in aesthetic appreciation, fashion and art, or in social statuses and more. Some peculiar accentuation defines for example professions, ranks, military grades, or the uniqueness of an individual like the crown of a king or the white cassock wearable only by Pope. All of these accents create illusions useful to rule human life, social interactions, and psychological status.

**Figure 17:**
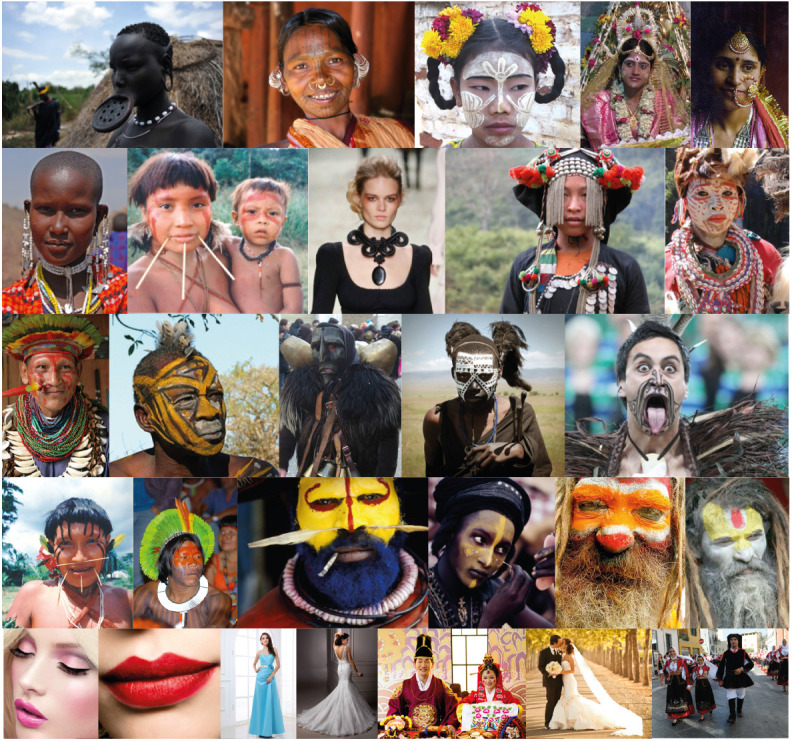
The complexity of human life is strongly related to the creation of illusions mostly through accents of expressive attributes affecting significantly different ethnic groups, cultures, historical periods, sexual dimorphism, and so forth.

Figure 17 demonstrates the complexity of these effects in different ethnic groups, cultures, historical periods, sexual dimorphism, and so forth. These accents create differences, switch inner implicit attributes, highlight explicit shapes and properties, induce new emerging meanings similar to the illusory creatures of Figure 14B. It cannot be denied that all of these accents are related mostly to the perceptual organization and, more importantly, are illusions strong and effective enough to shape human history and human life for good or for bad.

There is a further element to be considered within the biological domain. This is the movement, necessary for most living creatures, requiring the creation and use of illusions. As shown in Figure 14B, the location of the accents at the antipodes of the head induces an illusory motion direction. This is an effective tool to induce a deceptive illusion. The eyespots depicted in the liveries of fishes of Figure 14B may be evolved to reflect the shape of their body and to reverse the directions of the perceived motion.

To prove this we should first demonstrate that perceptual organization can cause a strong apparent motion. This is the topic of the next section.

### Apparent motion from grouping

Actually, a slight illusory motion can be seen in the patterns of Figure 1-second row. This result appears like a sliding effect due to the segregation of the elements from their background and to their elevation in depth that elicits some kind of floating motion. By shaking the stimulus, the floating motion increases. More particularly, in the patterns of Figures 1Af,g, the inset sub-matrixes appear floating respectively closer or further than the surrounding elements. More generally, the elements with white boundaries are perceived as floating closer to the observer than those fully black (Figure 1Ah).

**Figure 18:**
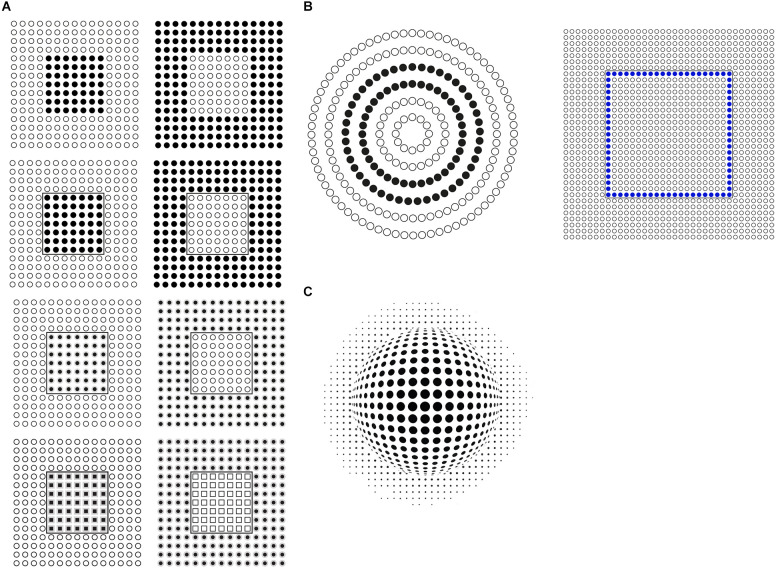
**(A)** Apparent floating motion increases with increasing the strength of the figure-ground segregation imparted by the similarity/dissimilarity of the inset/surrounding elements. **(B)** Apparent floating motion of the filled elements. **(C)** Apparent floating motion of the emerging sphere.

As illustrated in Figure 18A, the floating motion effect increases with increasing the strength of the figure-ground segregation (Rubin, 1921; Pinna and Spillmann, 2005) imparted by the similarity/dissimilarity of the inset/surrounding elements of the arrays.

The disconnection of the elements due to the similarity/dissimilarity among the two groups of filled and empty dots and the resulting floating motion of the black/blue dots are shown in Figure 18B. The inset dissimilar elements are perceived as floating irregularly as the gaze or the head is moved around the stimuli or, alternatively, by shaking the patterns.

In Figure 18C, a similar phenomenon is also perceived on the sphere, filled with dots on its surface, emerging from the background of distant elements. The floating phenomenon appears now moving less randomly and more related to the 3D organization of the dots.

**Figure 19:**
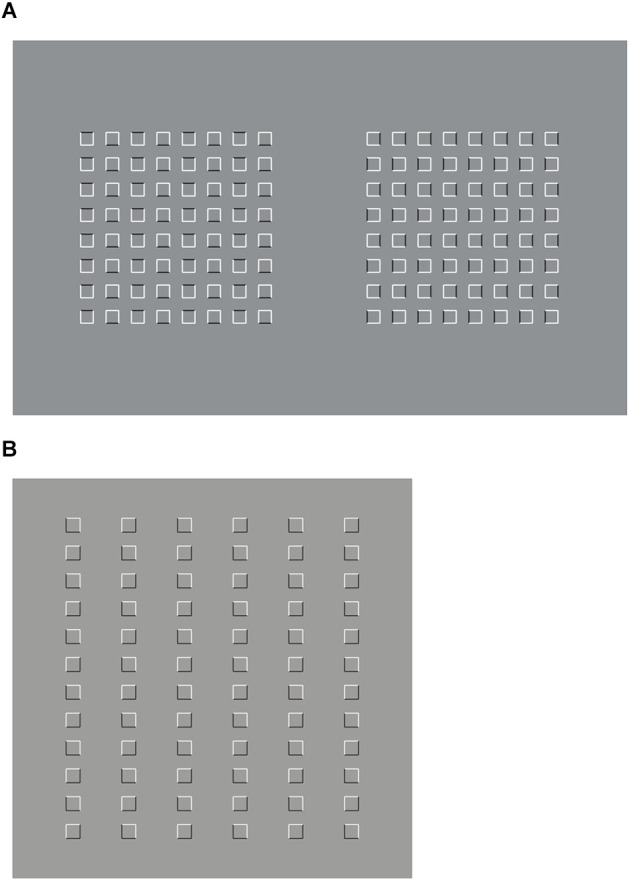
**(A)** The checks seem to go up and down. **(B)** Floating motion of the checks perceived as rhombic shapes or as stretched squares on opposite diagonals.

Further demonstrations of the role of perceptual grouping in eliciting directional organization, shape deformations, and sliding effects are illustrated in Figures 19A,B. More particularly, in Figure 19A, only one side of each geometrical square has been reversed in contrast, black against the white of the other sides.

Phenomenally, the checks appear statically going up and down and left and right respectively in the two patterns of Figure 19A. They are static and the dynamic effect is mostly a tendency to move than a real apparent motion, however, some kind of indeterminate visible motion among columns and rows can be seen. This effect emerges more easily when the gaze follows the tip of a pen moving vertically or horizontally across the checks but with attention and peripheral vision focused on the surrounding elements. While the pen is moving vertically the sliding motion is more easily perceived horizontally and, *vice versa*, when the pen moves horizontally the sliding motion occurs vertically. In addition, the rectangle illusion, previously described, can also be perceived.

The shape distortion and the apparent motion are more strongly perceived in Figure 19B, where the reversed contrast involves two opposite angles of each check that are again opposite to those of the adjacent checks. Instead of the rectangular deformation, the checks are now perceived as rhombic shapes or as stretched squares on opposite diagonals. This effect is much stronger in peripheral vision. Moreover, by using the previous technique based on fixing the tip of a pen moving vertically or horizontally, the apparent motion in opposite directions, horizontally or vertically, is promptly perceived.

**Figure 20:**
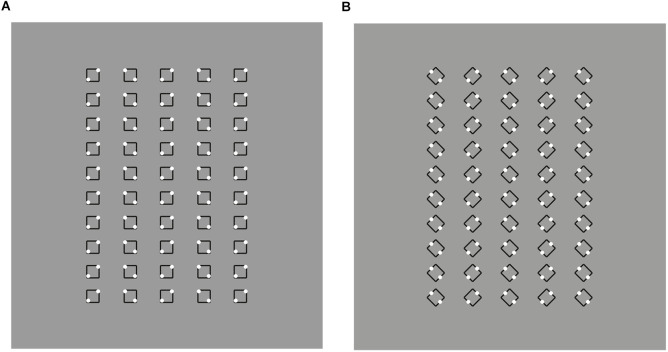
**(A)** When the gaze follows the tip of a pen moving vertically or horizontally across the checks but with attention and peripheral vision focused on the surrounding elements, a clear sliding motion is perceived. **(B)** Rectangle illusion and sliding motion.

A slight deformation of the checks and sliding motion are still perceived when the angles of the checks are accentuated by white dots, as shown in Figure 20A.

Now, by tilting the square check 45° and accentuating the sides instead of the angles, both rectangle illusion and sliding motion increase their strength (Figure 20B). It seems impossible that, by mentally translating one check until it matches the adjacent check with opposite accentuated directions, the two overlapped rectangles can appear as equal. Rather, they seem as two rectangles placed orthogonally.

**Figure 21:**
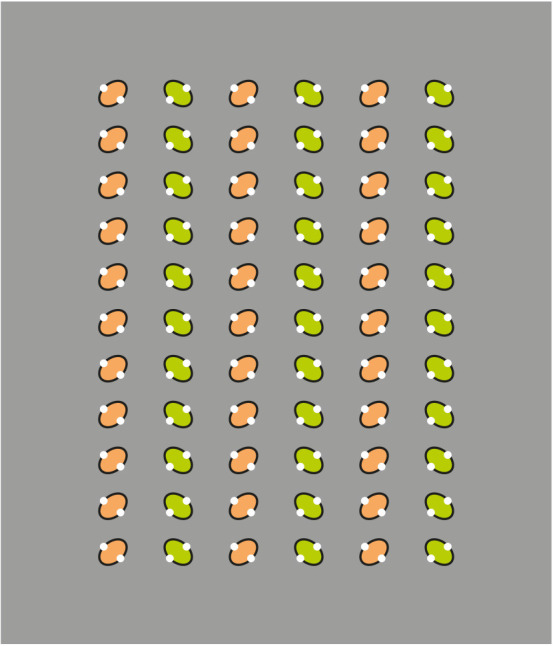
A very powerful sliding motion.

The apparent motion is now much stronger and perceived even without the use of the pen in Figure 21.

The results of this section support the role of grouping principles in inducing strong visual illusions with a very prominent illusoriness in the last conditions. In such cases, illusoriness is suggested from the awareness of the connection between the apparent motion and the true motion of the pen, of the observer, or of the stimulus. In other terms, the perceived motion appears to be related to the motion of the observer. Different from the real world, where objects and observers are commonly independent. Here, the motion of the elements is entangled with one of the observers. This is a strange, unexpected phenomenon generating strong surprise and astonishment. This is the main source of the illusoriness as opposed to realness.

## In The End Is Perceptual Organization

### Similarity can destroy illusions

**Figure 22:**
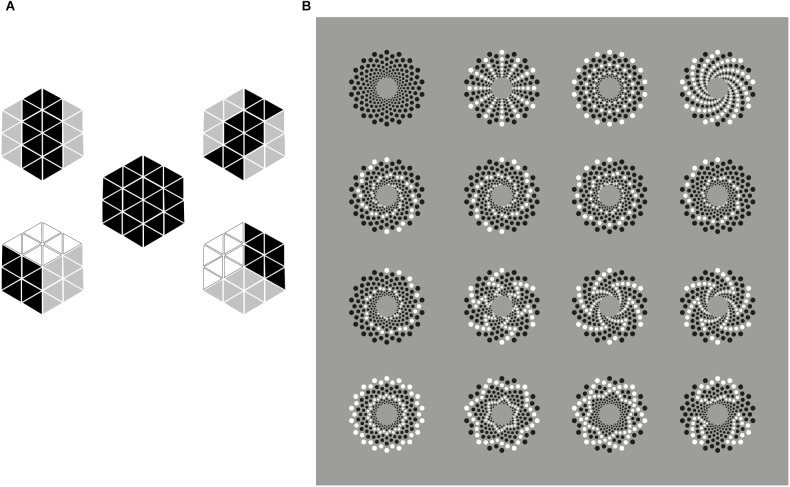
**(A)** The central hexagon is reorganized to be perceived as different objects: hexagons or cubes otherwise tilted. **(B)** The first pattern made up of black elements is reorganized by the principle of similarity in different ways against the proximity principle.

In all the previous sections, we demonstrated the causal role of similarity as a useful tool to reorganize patterns of elements by highlighting invisible objects and eliciting visual illusions. In Figures 22A,B, this role is further reinforced. More particularly, in Figure 22A, the central hexagon is reorganized to be perceived as different objects: hexagons or cubes otherwise tilted. In Figure 22B, the first pattern made up of black elements is reorganized in many ways against the proximity principle.

**Figure 23:**
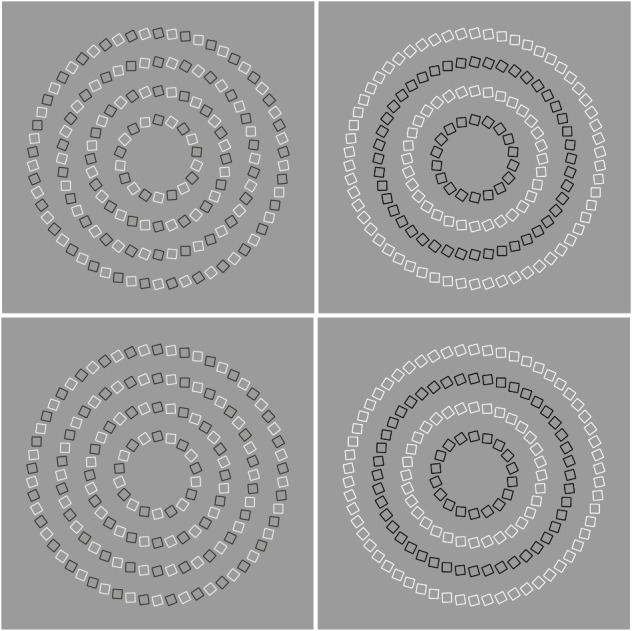
Grouping by similarity can destroy illusions. The dissimilarity of the checks on the left shows respectively a double intertwined spiral and a spiral (first column). These phenomena almost disappear when the similarity groups the elements highlighting the concentric rings of slightly tilted squares.

Nevertheless, grouping by similarity can also destroy illusions. In Figure 23, the dissimilarity of the checks shows respectively a double intertwined spiral and a spiral, in the first column. These compelling phenomena almost disappear when the similarity groups the elements highlighting the concentric rings of slightly tilted squares as shown.

This outcome only apparently is in contradiction with our rationale. As a matter of fact, it demonstrates that grouping principles are much more than generator of groups. They do not answer only the starting Wertheimer’s questions: “how do individual elements “go together” to form a holistic percept? How do wholes are perceived starting from single discrete elements?” They are much more effective in eliciting a high number of different phenomena.

Our previous results and, more particularly, the one shown in Figure 23, suggests that, on one hand, grouping principles are useful to generate and explain visual illusions, on the other hand, visual illusions can be useful to understand grouping and, more generally, perceptual organization.

## Conclusions

### The reality of illusions

In this work, we explored the notion of illusion starting from the principles of perceptual organization as described by Gestalt psychologists. On the basis of several phenomenal conditions, step by step, we suggested some new hypotheses, whose purpose was to answer the following questions: What is physical, and what is phenomenal? Is there and, if any, what is the dividing line between illusions and non-illusions? Is it true that illusions are rare phenomena? Why do illusions exist? What is their perceptual and evolutionist role?

These questions and the related issues were phenomenally discussed by deepening and extending the notion of perceptual organization and by exploring the biological implications of both illusions and illusoriness.

Perceptual organization and, more particularly, the principle of similarity was demonstrated to induce strong visual illusions. This principle does not merely put together discrete elements, but, by grouping them, creates segregations, highlight directions, shapes, attributes, and orientations and elicit apparent motion. In short, this principle and, more generally, perceptual organization induce compelling visual illusions than the other way around, were demonstrated to be useful to better understand the complex dynamics and expressiveness of visual organization much beyond the classical Gestalt principles of grouping and figure-ground segregation. This is the reality of illusions.

### The illusion of reality

Our results suggested that there is not any dividing line between perceptual organization and illusions, i.e., between non- illusions and illusions. This statement does not imply that everything is an illusion. The risk is, in fact, to fall into the conclusion that if everything is an illusion nothing is. More interestingly, we suggest that the notion of illusion is an effective and short route to explore the unexplored territory of the ways of seeing, source of different, sometimes controversial, and antinomic outcomes that we are able to perceive, compare, and meta-perceive. This is where illusions and illusoriness come out. Illusions are always the result of a discovery based on comparisons among ways of seeing, some of them useful to get into the geometrical/physical domain, some to explore different layers of what we called “gradient of phenomenalness,” others necessary to compare different and opponent results that let us discover illusions.

This entails that illusions are attributes of visual objects to be discovered, much like the visual process of discovering a frog totally camouflaged in the background of a tree bark. Therefore, it could be correct to state that everything is an illusion, however, nothing is illusory if we, first, do not discover it, and to discover illusions different ways of seeing are necessary. The camouflaged frog is not an illusion if we do not discover and perceive it. In this case, the frog does not exist. This entail that the conclusion “if everything is an illusion nothing is” is incorrect mostly in the second part of the statement “nothing is an illusion.” The first part could be, instead, correct. Everything can be an illusion if we are able to unveil and demonstrate it.

### Illusions as biological requiredness

The crucial point of our demos is the following: if grouping principles are, at the same time, generators of illusions, then illusions should be the basic component of the perceptual world of the living beings. In other words, since illusions are spontaneously and immediately created by the basic principles of organization, then, through adaptation and natural selection, they can be used by living beings for multiple survival purposes. Thus, evolution can develop more and more effective ways to take advantage for survival and adaptation purposes of the properties of the perceptual organization to generate illusions. By deeply reflecting on this point, we can realize that most of the natural life of living organisms is based on the ability to create illusions to deceive, attract, intimidate, emerge, disappear, hide and show. All of these illusory adaptations, evolutions, behaviors, and appearances are illusions. However, they are illusions only when they are discovered and unveiled becoming aware of the multiple outcomes delivered by different ways of seeing, e.g., when a camouflaged predator ambush is discovered by a prey or, *vice versa*, when a predator discovers the deception carried out by a prey. Within the domain of the human species, deceptions through illusions are even more essential part of our everyday life, for example through fashion, make-up, accentuations of power, strength, intelligence, social status, and so on.

In short, we suggest that illusions are not rare and niche phenomena, quite the opposite, illusions can be considered as true biological requiredness of natural selection and Darwinian fitness for all living organisms and this is because they are immediate outcomes of perceptual organization and of the grouping principles.

### Consciousness from illusoriness

There is a further point, explored in this work, full of promising consequences. Some of the phenomena demonstrated an intense sense of illusoriness, perceived as something having the nature of an illusion, something unreal, ambiguous, fallacious, deceptive, false, fictitious, or misleading. This attribute emerges through the visual comparison of outcomes delivered by different ways of seeing and is perceived with different degrees of vividness, sometimes weakly, in other cases very strongly and independently from any mismatch between domains.

According to the idea of illusions as biological requiredness, the perception of illusoriness attribute is related to the visual consciousness of multiple outcomes within the same stimuli. These outcomes derive from different ways of seeing supervised by a meta-perceptual way of seeing, that is visual consciousness aimed to make simple or crucial decisions like, for example, to attack or give up in the case of a predator, or to run away or stay still in the case of a prey.

On the basis of our results, the perception of illusion and illusoriness can be considered as a further challenge for vision scientists useful to shed new insights within the biological meanings of visual perception and within the no-man land between sensory and cognitive processes that elicit visual consciousness not fully explored yet.

## Data Availability Statement

The raw data supporting the conclusions of this article will be made available by the authors, without undue reservation.

## Ethics Statement

Ethical review and approval was not required for the study on human participants in accordance with the local legislation and institutional requirements. Written informed consent from the participants was not required to participate in this study in accordance with the national legislation and the institutional requirements.

## Author Contributions

BP, DP, and JS contributed to the conception, study design, and theoretical analysis. All authors critically reviewed the manuscript and contributed to the editing of the final draft. All authors contributed to the article and approved the submitted version.
